# Preliminary Checklist for Reporting Observational Studies in Sports Areas: Content Validity

**DOI:** 10.3389/fpsyg.2018.00291

**Published:** 2018-03-08

**Authors:** Salvador Chacón-Moscoso, Susana Sanduvete-Chaves, M. Teresa Anguera, José L. Losada, Mariona Portell, José A. Lozano-Lozano

**Affiliations:** ^1^HUM-649 Innovaciones Metodológicas en Evaluación de Programas, Departamento de Psicología Experimental, Facultad de Psicología, Universidad de Sevilla, Sevilla, Spain; ^2^Departamento de Psicología, Universidad Autónoma de Chile, Santiago de Chile, Chile; ^3^Faculty of Psychology, Institute of Neurosciences, University of Barcelona, Barcelona, Spain; ^4^Department of Psychobiology and Methodology of Health Sciences, Universitat Autònoma de Barcelona, Cerdanyola del Vallès, Spain

**Keywords:** checklist, reporting, observational studies, sports area, content validity, experts, Osterlind index

## Abstract

Observational studies are based on systematic observation, understood as an organized recording and quantification of behavior in its natural context. Applied to the specific area of sports, observational studies present advantages when comparing studies based on other designs, such as the flexibility for adapting to different contexts and the possibility of using non-standardized instruments as well as a high degree of development in specific software and data analysis. Although the importance and usefulness of sports-related observational studies have been widely shown, there is no checklist to report these studies. Consequently, authors do not have a guide to follow in order to include all of the important elements in an observational study in sports areas, and reviewers do not have a reference tool for assessing this type of work. To resolve these issues, this article aims to develop a checklist to measure the quality of sports-related observational studies based on a content validity study. The participants were 22 judges with at least 3 years of experience in observational studies, sports areas, and methodology. They evaluated a list of 60 items systematically selected and classified into 12 dimensions. They were asked to score four aspects of each item on 5-point Likert scales to measure the following dimensions: representativeness, relevance, utility, and feasibility. The judges also had an open-format section for comments. The Osterlind index was calculated for each item and for each of the four aspects. Items were considered appropriate when obtaining a score of at least 0.5 in the four assessed aspects. After considering these inclusion criteria and all of the open-format comments, the resultant checklist consisted of 54 items grouped into the same initial 12 dimensions. Finally, we highlight the strengths of this work. We also present its main limitation: the need to apply the resultant checklist to obtain data and, thus, increase quality indicators of its psychometric properties. For this reason, as relevant actions for further development, we encourage expert readers to use it and provide feedback; we plan to apply it to different sport areas.

## Introduction

Observational studies are mainly based on systematic observation, understood as an organized recording and quantification of behavior in its natural context (Anguera, 1979, 1996, 2003). These types of studies involve a low level of intervention (Chacón-Moscoso et al., 2013). Observational studies present the following important advantages compared to those with a medium or high level of intervention (Portell et al., 2015), such as: (1) they can be adapted to any situation in any setting and (2) they do not need standardized measurement instruments because the context of the study is prioritized and, as a consequence, the use of ad hoc instruments is accepted.

Observational studies are commonly used in many areas, such as social (Anguera and Redondo, 1991; Santoyo and Anguera, 1992), psycho-pedagogical (Moya et al., 2012; Herrero-Nivela et al., 2014), clinical (Roustan et al., 2013; Arias et al., 2015), or sport (Weigelt and Memmert, 2012; Anguera and Hernández-Mendo, 2014) studies. The concrete field of observational methodology, as applied to sports, currently benefits from the advanced development of statistical analyses and specific software to study men and women's sports-related behaviors in order to obtain indicators to improve their performance (Anguera and Hernández-Mendo, 2015; Anguera et al., 2017, 2018). For example, (1) sequential analysis of behaviors using SDIS-GSEQ software (Bakeman and Quera, 2011) has been developed to establish model sequences by high-level sportsmen and women (Castelão et al., 2015); (2) the use of polar coordinate analysis by means of HOISAN software (Hernández-Mendo et al., 2012) enables the study of interrelations between different categories of observational tools in different sports, such as tae kwon do (López-López et al., 2015), handball (Sousa et al., 2015), or soccer (Castellano and Hernández-Mendo, 2003; Castañer et al., 2016); and (3) T-pattern analysis using Theme software (Magnusson, 1996, 2000) can be applied to discover hidden structures in the observed behavior that are not directly visible in elite climbing (Arbulu et al., 2016), futsal (hard-court soccer, Sarmento et al., 2016), synchronized swimming (Iglesias et al., 2015), or bouts epee (fencing, Tarrag et al., 2015).

Although observational studies are frequently used and their utility in different contexts has been widely proven, a tool to measure the reporting quality of these types of studies does not exist, nor does a specific one for sports areas (Portell et al., 2015). This lack causes important consequences for observational studies in sports areas: (1) the author's report is the unique information we usually have about primary studies (Altman et al., 2001; Grimshaw et al., 2006; Cornelius et al., 2009). As authors do not have a checklist for reporting, transparency may be affected, and important information for assessing the quality of the study and, therefore, its degree of risk of bias, may be omitted (Portell et al., 2015). (2) Authors who want to publish these kinds of studies do not have a checklist to confirm that all the important elements were considered in the study and included in the report, and reviewers of these same studies lack a useful tool for determining the indicators to consider when accepting or rejecting their publication in a scientific journal (Chacón-Moscoso et al., 2016).

Checklists to measure the quality of the reporting of primary studies in general, without specifying the design type, have previously been published, e.g., by the Journal Article Reporting Standards (JARS) (American Psychological Association, 2010). In addition, as a consequence of the differences existing across designs, checklists with the same purpose but for specific study designs have been published (Portell et al., 2015; Chacón-Moscoso et al., 2016). For example, for high-intervention designs (randomized control trials), we have the Consolidated Standards of Reporting Trials (CONSORT) (Schulz et al., 2010); for epidemiological studies, such as cohort, case-control, and cross-sectional studies, we have the Strengthening the Reporting of Observational Studies in Epidemiology (STROBE) statement (von Elm et al., 2007); (3) for intensive repeated measurements in naturalistic settings, we have the Guidelines for Reporting Momentary Studies (Stone and Shiffman, 2002); (4) for qualitative studies, we have the Guidelines for Qualitative Research Methodologies (Blignault and Ritchie, 2009); and (5) for mixed methods, we have the Guidelines for Conducting and Reporting Mixed Research for Counselor Researchers (Leech and Onwuegbuzie, 2010). The current standard for low-intervention studies is the Guidelines for Reporting Evaluations Based on Observational Methodology (GREOM) (Portell et al., 2015; included in the EQUATOR library: http://www.equator-network.org/reporting-guidelines/guidelines-for-reporting-evaluations-based-on-observational-methodology/) which, combined with JARS, provides a general view of the structural characteristics of observational designs that must be considered for evaluation in low-intervention situations without specifying any concrete area.

The aim of this work is to develop a checklist to measure the quality of the reporting of sports-related observational studies. This checklist will further clarify the general guidelines presented in the GREOM; determine the main quality indicators of the reporting of sports-related observational studies; serve as a useful tool for authors conducting and publishing observational studies in this area, as well as for reviewers making decisions for publications; and present indicators of the representativeness (REP), relevance (REL), utility (U), and feasibility (F) of the developed tool to measure the quality of the reporting based on a content validity study.

## Methods

### Participants

Twenty-two of the 43 potential candidates who were contacted opted to participate in the study, resulting in a participation rate of 51.2%. The inclusion criteria for eligibility were a minimum of 3 years of experience in observational studies, sports areas, and methodology. The sample participants were between 31 and 70 years old [mean (*M*) = 45.9, standard deviation (*SD*) = 11.3], including 17 men (77.3%) and 5 women (22.7%). Their years of experience in methodology were between 3 and 44 (*M* = 17.1, *SD* = 11.7). Their years of experience in observational studies ranged from 3 to 35 (*M* = 13.6, *SD* = 9.3). Finally, their years of experience in sports areas ranged between 3 and 40 (*M* = 19.9, *SD* = 10).

The main dedication and sports interest of these experts was in the area of physical education (8 participants, 36.4% of the sample), understood as the set of disciplines that aim to develop the human body through sports participation and encourages psychomotor learning in a game-like setting or through movement exploration. This is a commonly introduced subject in primary and secondary education curricula (Woodward, 2016). The second most frequent area of interest was high-performance sports (five participants, 22.7%), referring to the type of activity (individual and team) in competition contexts (Harenberg et al., 2016). The third area of interest was sport initiation (4 participants, 18.2%), the process by which a person makes contact with new experiences in a physical activity or sport (Thomas et al., 2015). The areas of health, sports, and physical education (a set of educational, sporting, and organizational practices to promote well-being and health; Williams and Macdonald, 2015) and adapter sports (sports practices of people with some kind of physical and/or psychological disability; Park and Sinelnikov, 2016) were chosen by two participants each (9.1%). Finally, one participant (4.5%) chose technology, defined as the tools aimed to improve athletes' sports performance in order to set personal records and, thus, be more competitive (Hardcastle et al., 2015).

### Instruments

Appendix I in Supplementary Material presents the instrument we designed to enable content validity experts to determine the main aspects of sports-related observational studies. It is composed of 60 items representing 12 dimensions from the GREOM (Portell et al., 2015): (1) *Extrinsic characteristics* (1 item); (2) *Objectives delimitation* (6 items); (3) *Observational design* (3 items); (4) *Participants* (9 items); (5) *Context-setting* (11 items); (6) *Observational instrument* (7 items); (7) *Recording instrument* (6 items); (8) *Data* (3 items); (9) *Parameter specification* (2 items); (10) *Observational sampling* (6 items); (11) *Data quality control* (5 items); and (12) *Data analysis* (1 item).

For the content validity study, four 5-point Likert scales (Sanduvete-Chaves et al., 2013) were associated with each item to be assessed by the experts referring to four different aspects with respect to its dimension: (1) REP referred to the degree to which each item represented the dimension to which it had been assigned; (2) REL was defined as the extent to which each item was important or highlighted something of the dimension in which it was included; (3) U referred to the extent to which each item was useful to evaluate the dimension to which it was assigned; and (4) F was defined as the possibility of recording information about each item. Additionally, an open-format item (comments) was available for experts who wished to propose something new, such as improving the writing of an item or exchanging it for something more appropriate.

This instrument was available in two formats: the Internet format using Google Drive Forms and a paper version. Microsoft Excel was used for the data analysis.

### Procedure

Ethical approval and written informed consent were not needed for our study, as the participants were experts, a non-vulnerable group, and the information gathered was professional opinions about the adequacy of different items used to report observational studies, without medical, clinical, or personal implications.

#### Item selection and assignment to dimensions

We delimited the main dimensions of observational studies and a list of items to measure those dimensions based on three information sources: (1) A systematic review (Chacón-Moscoso et al., 2016) was conducted of 12 databases that were of interest due to their content (Web of Science, Scopus, Springer, EBSCO Online, Medline, CINAHL, EconLit, MathSciNet, Current Contents, Humanities Index, ERIC, and PsycINFO). We found 548 different ways to measure methodological quality in primary studies. From this total, some of the tools were general reporting standards not specific to any particular research design (e.g., Zaza et al., 2000; American Educational Research Association, 2006; American Psychological Association, 2010; Möhler et al., 2012), while others were specific reporting standards for research designs with some similarities to observational designs (Stone and Shiffman, 2002; Tong et al., 2007; Blignault and Ritchie, 2009; Pluye et al., 2009; Leech and Onwuegbuzie, 2010). (2) The GREOM (Portell et al., 2015) represents the specific guidelines for developing observational studies. As an illustrative example of the GREOM's high degree of influence over the list of items gathered, apart from the common structure, we can see the direct correspondence in dimensions of the present checklist 6 *Observational instrument*, 7 *Recording instrument*, and 9 *Parameters specification*, according to section B2 *Instruments* of GREOM, including the guidelines 7 *Observation instrument*, 8 *Primary recording parameters*, and 9 *Recording instruments*. (3) The final dimension involves sports-related observational studies found in the previously cited databases (Anguera and Hernández-Mendo, 2015).

Two coders separately studied the degree of agreement in the items dimension assigned and intercoder reliability (Nimon et al., 2012; Stolarova et al., 2014) was studied by calculating Cohen's κ (Cohen, 1960). Any disagreements were resolved by consensus.

#### Content validity study

Once the 60 items were selected and assigned to one of the 12 dimensions chosen, the experts were asked, through 5-point rating scales, about the REP, REL, U, and F of each item with respect to its dimension.

The instrument was sent to experts in English (Appendix I in Supplementary Material) or Spanish (Supplementary Material), depending on their native language. We provided the access link to the instrument in Google Drive by sending an email to the potential experts that satisfied the participant's inclusion criteria. Fifteen days later, we reminded the participants that the instrument was available to be fulfilled in the same link. After another 15 days, we made the last call for answers in the same way. After a final 15 days, the application was definitively closed. As part of the final gathering stage, the same instrument was available in paper format for all of the participants (who worked in observational studies, methodology, and sports areas) at the VII European Congress of Methodology, held in Palma de Mallorca (Spain) in July 2016. Throughout the entire process, the information was gathered anonymously.

After gathering the information, the Osterlind index of congruence (Osterlind, 1998) was calculated for each item and each aspect measured (REP, REL, U, and F). The formula used was.

$$\begin{array}{l} I_{ik}=\frac{\left(N-1\right)\overset{n}{\underset{j\;=\;1}{\sum }}X_{ijk}+N\overset{n}{\underset{j\;=\;1}{\sum }}X_{ijk}-\overset{n}{\underset{j\;=\;1}{\sum }}X_{ijk}}{2\left(N-1\right)n} \end{array}$$

where *N* = number of dimensions (12 in this case), *X*_*ijk*_ = score provided by each expert to each item referred to each aspect measured, and *n* = number of experts. The scores were provided in a 5-point Likert scale (−1 = strongly disagree, −0.5 = disagree, 0 = neither agree nor disagree, 0.5 = agree, and 1 = strongly agree) instead of the classical one with 3 points, to make the achievement of high results slightly difficult, as the 5-point version is more conservative (Revised Osterlind Index, Sanduvete-Chaves et al., 2013).

The results of the previous formula ranged from −1 to +1. Minus one implied a total agreement among the experts, indicating that all answered that they disagreed strongly; 1 meant a total agreement among the experts, positioning all in strong agreement; and 0 represented the highest possible disagreement among the experts.

Based on the criteria (Osterlind, 1998), items that obtained a score of 0.5 or higher on the four aspects measured were included in the final version of the checklist for reporting observational studies.

## Results

The assignment of the 60 items selected to the 12 dimensions made by two independent researchers obtained a degree of consensus of κ = 0.76 (*p* < 0.001) and a 95% confidence interval (CI) of [0.646, 0.874]. This result can be considered appropriate (Landis and Koch, 1977).

Forty-three experts were contacted by email to fulfill the content validity questionnaire. A total of 14 experts answered via Google Drive. Two participants sent their responses after the first call for answers, five participants answered in the second round, and seven additional experts gave their opinions in the final round. Additionally, eight experts fulfilled the questionnaire in paper format during the VII European Congress of Methodology (July 2016). The total number of answers gathered was 22. According to Prieto and Muñiz (2000), a number of experts ranging from 10 to 30 through a systematic procedure can be considered a moderate sample size.

Table 1 presents the Osterlind indexes obtained for each item referring to REP, REL, U, and F. Fifty-three items met the criterion of having a result of 0.5 or higher in these four aspects. Only seven items were removed because they did not meet this criterion: those in dimension 4 referred to the participants, items 12 (cultural background), 13 (socio-economic level), 17 (differential exclusion of participants), and 18 (participants' allocation); those in dimension 5 referred to the context (setting), item 24 (number of non-observable periods); those in dimension 6 referred to the observational instrument, item 34 (criteria that lead to the catalogs and categories systems); and those in dimension 9 referred to the parameters specification, item 48 (parameters fitting). The removed items appear in bold text in Table 1.

**Table 1:** Osterlind indexes obtained for each item in representativeness (REP), relevance (REL), utility (U), and feasibility (F).

**Item^a^**	**REP**	**REL**	**U**	**F**
**DIMENSION 1. EXTRINSIC CHARACTERISTIC**					
1. Publication type	0.90	0.93	0.94	0.93
**DIMENSION 2. OBJECTIVES DELIMITATION**					
2. Problem delimitation, sport	0.77	0.93	0.84	0.85
3. Problem delimitation, general objectives	0.93	1	0.95	0.93
4. Problem delimitation, specific objectives	0.93	0.90	0.88	0.85
5. Reference to theoretical framework	0.71	0.81	0.79	0.74
6. Specification of response levels	0.82	0.83	0.80	0.78
7. Specification of participation degree	0.70	0.76	0.70	0.73
**DIMENSION 3. OBSERVATIONAL DESIGN**					
8. Specification of observational design for each objective	0.86	0.83	0.93	0.90
9. Justification of the observational design	0.81	0.79	0.73	0.73
10. Sequence data are obtained	0.50	0.79	0.70	0.60
**DIMENSION 4. PARTICIPANTS**					
11. Age	0.50	0.58	0.66	0.66
**12. Cultural background**	**0.34**	**0.21**	**0.33**	**0.33**
**13. Socio-economic level**	**0.30**	**0.36**	**0.33**	**0.28**
14. Sport modality	0.55	0.64	0.65	0.80
15. Professionalism	0.60	0.62	0.60	0.58
16. Global exclusion of participants	0.66	0.68	0.58	0.60
**17. Differential exclusion of participants**	**0.57**	**0.40**	**0.42**	0.52
**18. Participants' allocation**	**0.57**	**0.50**	**0.42**	**0.40**
19. Activity type	0.68	0.62	0.68	0.75
**DIMENSION 5. CONTEXT (SETTING)**					
20. Place (location)	0.61	0.62	0.63	0.83
21. Social impact of the activity	0.59	0.52	0.53	0.63
22. Time frame	0.66	0.67	0.73	0.75
23. Session acceptance criteria	0.68	0.64	0.70	0.65
**24. Number of non-observable periods**	0.66	0.55	0.55	**0.45**
25. Duration of non-observable periods	0.73	0.71	0.75	0.73
26. Total results indication	0.73	0.69	0.65	0.68
27. Partial results indication	0.59	0.69	0.65	0.63
28. Observational unit adjustment	0.61	0.69	0.65	0.60
29. Observational units delimiting, denominating and definable	0.70	0.71	0.73	0.68
30. Global/molecular units' degree justified	0.91	0.95	0.93	0.88
**DIMENSION 6. OBSERVATIONAL INSTRUMENT**					
31. Type of observational instrument	0.86	0.79	0.82	0.73
32. Instrument appropriate to the design	0.80	0.74	0.68	0.75
33. Justification of instrument type according to the observational design	0.66	0.69	0.65	0.68
**34. Criteria that led to the catalogs and categories systems**	0.64	**0.43**	0.55	0.50
35. Requirements to categorize from a certain criterion	0.64	0.60	0.55	0.63
36. Availability of full coding manual	0.80	0.79	0.68	0.75
37. Observational instrument adequate to the study context	0.75	0.74	0.75	0.78
**DIMENSION 7. RECORDING INSTRUMENT**					
38. Software utilization as user	0.70	0.68	0.70	0.65
**DIMENSION 8. DATA**					
39. Software type used to record	0.68	0.64	0.63	0.70
40. Observational recording	0.80	0.81	0.75	0.80
41. Software used to record	0.91	0.93	0.98	0.93
42. Software used for data quality control	0.91	0.90	0.93	0.90
43. Software used for data analysis	0.82	0.83	0.85	0.83
44. Type of data according to Bakeman (1978)	0.75	0.62	0.68	0.75
45. Type of data according to Bakeman (1983)	0.70	0.60	0.55	0.65
46. Data management	0.66	0.60	0.53	0.53
**DIMENSION 9. PARAMETER SPECIFICATION**					
47. Parameter type (the most complex)	0.64	0.52	0.55	0.50
**48. Parameter fitting (the most complex)**	**0.48**	**0.48**	**0.45**	0.53
**DIMENSION 10. OBSERVATIONAL SAMPLING**					
49. Observational period	0.75	0.57	0.68	0.74
50. Sessions periodicity	0.75	0.60	0.70	0.73
51. Number of sessions	0.86	0.71	0.78	0.74
52. Starting session criterion	0.75	0.76	0.68	0.68
53. Ending session criterion	0.80	0.74	0.73	0.78
54. Within-session sampling	0.70	0.57	0.65	0.58
**DIMENSION 11. DATA QUALITY CONTROL**					
55. Agreements	0.67	0.60	0.58	0.50
56. Concordance	0.93	0.93	0.90	0.85
57. Within-session reliability	0.86	0.79	0.80	0.75
58. Between-session reliability	0.84	0.76	0.78	0.75
59. Generalizability theory application	0.76	0.70	0.71	0.66
**DIMENSION 12. DATA ANALYSIS**					
60. Data analysis developed	0.98	0.90	0.89	0.89

*REP, representativeness; REL, relevance; U, utility; F, feasibility. One item is considered appropriate when the values obtained in the four aspects measured (REP, REL, U, and F) are at least 0.5. We marked the Osterlind indexes under 0.5 and removed the items in bold text*.

a *Items appear in abbreviated form; the full items can be consulted in Appendix I (Spanish version in Supplementary Material)*.

Analyzing all of the items as a whole and taking into account that the possible results ranged from −1 to 1, we found that, in REP, *Mdn* = 0.71 (*SD* = 0.14), with the values ranging from 0.3 to 0.98; in REL, *Mdn* = 0.69, *SD* = 0.16, range = 0.21–1; in U, *Mdn* = 0.69, *SD* = 0.15, range = 0.33–0.98; finally, in F, *Mdn* = 0.73, *SD* = 0.14, range = 0.28–0.93.

Table 2 presents the open-format comments made by the experts and the actions developed in order to follow their advice. From a total of 22 different comments, all were followed with the exception of one (item 11), to which we made only a partial change. Four comments did not imply changes because they referred to items excluded by the Osterlind index results.

**Table 2:** Open-format comments provided by experts and actions taken as a consequence.

**Item^a^**	**Comment**	**Action^b^**
**DIMENSION 2. OBJECTIVES DELIMITATION**		
2. Problem delimitation, sport	Remove the alternative *psychomotor skills* and add *modified/reduced sport games*	Done
3. Problem delimitation, general objectives	Introduce one more option between *no* and *yes*	We introduced the intermediate option *yes, but unclearly*
4. Problem delimitation, specific objectives	Introduce one more option between *no* and *yes*	We introduced the intermediate option *yes, but unclearly*
5. Reference to theoretical framework	Remove the alternative *Regulation*	Done
6. Specification of response levels	Introduce one more option between *no* and *yes*	We introduced the intermediate option *yes, but unclearly*
**DIMENSION 3. OBSERVATIONAL DESIGN**		
9. Justification of the observational design	Introduce one more option between *no* and *yes*: *partial/incomplete*	Done
**DIMENSION 4. PARTICIPANTS**		
11. Age	Establish 10-year intervals from 19s	Based on basketball classification, we included one more category (*under 20s*). We did not include more intervals because, in the absolute category (senior), there is usually no age restriction
12. Cultural background	Introduce one more option between *no* and *yes*	Removed due to Osterlind index results
13. Socio-economic level	Introduce one more option between each pair of alternatives	Removed due to Osterlind index results
14. Sport modality	Related to this, add a new item: opposition (yes/no)	Done
15. Professionalism	Include the options *amateur* and *retired from professional sport*	Done
16. Global exclusion of participants	Include it in dimension 11, Data quality control	Done
17. Differential exclusion of participants	Include it in dimension 11, Data quality control	Removed due to Osterlind index results
18. Participants' allocation	It is not applicable in most observational studies. Include it in dimension 11, Data quality control	Removed due to Osterlind index results
19. Activity type	Include *physical exercise*	Done
**DIMENSION 5. CONTEXT (SETTING**		
21. Social impact of the activity	Change the options to low/medium/high	Done
28. Observational unit adjustment	Add the option *according to the player*	Done
**DIMENSION 6. OBSERVATIONAL INSTRUMENT**		
37. Observational instrument adequate to the study context	Include an intermediate option: *partial*	Done
**DIMENSION 7. RECORDING INSTRUMENT**		
41. Software used to record	Add *SportsCode*	Done
42. Software used for data quality control	Include this item in dimension 11 *Data quality control*	Done
43. Software used for data analysis	Include this item in dimension 12 *Data analysis*	Done

a *Items appear in abbreviated form; the full items can be consulted in Appendix I (Spanish version in Supplementary Material). Only the items that received some comment have been included in Table 2*.

b *Changes resulting from these comments are presented in the final version of the checklist (Appendix II)*.

All of the comments presented were provided by only one expert, except those referring to the graduation of the answers for some dichotomous items, which were proposed by five experts.

Appendix II in Supplementary Material presents the final version of the checklist for reporting sports-related observational studies after making the changes derived from the results of the Osterlind indexes and the experts' open-format comments. One proposal provided in open format was to add one more item. Originally, the instrument presented 60 items, and 7 were removed due to the Osterlind indexes, resulting in the inclusion of 54 items in the final version.

## Discussion

We propose a 54-item and 12-dimension checklist to measure the reporting quality of observational studies in sports areas. Its use by authors and reviewers may contribute to the increased transparency of these studies, as it lists the main aspects to consider and delimit when designing, executing, or evaluating observational studies in sports areas. The importance of this checklist resides in its exclusivity, considering that no other tool with this same purpose exists in the literature. There are other checklists available with the same objective as our proposal, that is, to measure the quality of reporting, although to be applied in other contexts (e.g., in orthopedics, Mundi et al., 2008) and other kinds of designs (e.g., in orthopedics and randomized control trials, Chan and Bhandari, 2007). Additionally, there are checklists in sports (e.g., Arnold and Schilling, 2017), but in designs different from observational studies (Anguera et al., 2018; as guidelines created for this methodology, readers can see the GREOM included in the EQUATOR library: http://www.equator-network.org/reporting-guidelines/guidelines-for-reporting-evaluations-based-on-observational-methodology/). On other occasions, we find checklists applied to similar designs (e.g., STROBE for epidemiological studies, von Elm et al., 2007), although not exactly for observational studies understood as an organized recording and quantification of behavior in its natural context.

These checklists present some characteristics in common with our proposal, such as the format (closed-ended questions) or the capacity to detect relevant information that has not been reported. Nevertheless, they differ in content, not only due to the sport context [e.g., item 14, *Sport modality: (1) Individual sport, (2) Team sport*; or item 15, *Professionalism: (1) Professionals, (2) Semi-professionals, (3) Sportsmen/women in training stage*], but also due to the casuistic of the observational design [e.g., item 9, *Justification of the observational design: (1) No, (2) Yes*; or item 24, *Number of non-observable periods*].

The main strength of this work is that the content validity study was developed through a clear, careful, and explicit process, so it presented a high degree of reproducibility. In this way, we were able to define a list of items based on different sources of information: a systematic review, the GREOM as the theoretical framework and the basis for the 12 delimited dimensions and content in several dimensions (illustrated in the correspondence between dimensions 6 *Observational instrument*, 7 *Recording instrument*, and 9 *Parameters specification* of the presented checklist and guidelines 7 *Observation instrument*, 8 *Primary recording parameters*, and 9 *Recording instruments*, corresponding to section B2 *Instruments* of the GREOM) (Portell et al., 2015), and published observational studies in sports areas. We provided the full list of items assessed by the experts in English (Appendix I in Supplementary Material) and Spanish (Supplementary Material). We determined the inclusion criterion a priori; we reported the Osterlind index for all of the items in the four aspects measured (Table 1). We objectively applied the previously established inclusion criterion and transcribed all of the open-format comments provided by the experts and each action we executed in answer to each comment (Table 2). After considering the Osterlind indexes and open-format comments, we presented the final version of the checklist for reporting sports-related observational studies (Appendix II in Supplementary Material).

Additionally, we obtained adequate results for the fitness item dimension with respect to four aspects: REP, REL, U, and F, which provides a quality indicator of the content validity in favor of the use of the resulting tool as appropriate. The resultant checklist is expected to be extensively useful, as it can be applied to any sports area.

On the other hand, the main limitation we found in the checklist obtained is that it supposes a preliminary proposal in which further development is needed to increase the quality indicators of its psychometric properties. For this purpose, we encourage and urge expert readers to improve our final version checklist (Appendix II in Supplementary Material) with their comments or results regarding its application.

Additionally, we plan to apply the checklist to different sports areas in order to demonstrate that it is an adequate measurement instrument independent of the sport context and to develop an intercoder reliability study to locate discrepancies across the independent coding of a high number of studies (more than 40) by two different previously trained coders. We consider this proposal as open and in progress, as we will continue to consider additional comments for the improvement of the checklist that we might receive by experts.

Taking this work as the basis, we plan to develop a scale to measure methodological quality in sports-related observational studies. This checklist can serve as a guideline for measuring the reporting quality of these studies because it lists the main aspects to consider when designing, executing, and evaluating a sports-related observational study. We can also recommend concrete actions to increase the methodological quality of these studies.

## Ethics statement

This study was carried out in accordance with the recommendations of the Declaration on bioethics and human rights, UNESCO, 2005 with written informed consent from all subjects. All subjects gave written informed consent in accordance with the Declaration of Helsinki. The protocol was approved by the Ethics Committee, Universidad Autónoma de Chile.

## Author contributions

All of the authors contributed to documenting, designing, drafting, and writing the manuscript, and revised it for important theoretical and intellectual content. Additionally, all of the authors provided final approval of the version to be published and agreed to be accountable for all aspects of the work in ensuring that questions related to the accuracy or integrity of any part of the work are appropriately investigated and resolved.

### Conflict of interest statement

The authors declare that the research was conducted in the absence of any commercial or financial relationships that could be construed as a potential conflict of interest.
