# Supplementary material for: Preliminary Checklist for Reporting Observational Studies in Sports Areas: Content Validity
Source: Front Psychol. 2018 Mar 8;9:291. doi: 10.3389/fpsyg.2018.00291 (PMC5853306; doi:10.3389/fpsyg.2018.00291)
Supplement: Supplementary file 1 [file Table1.docx]

Supplementary Material

Preliminary Checklist for Reporting Observational Studies in Sports Areas: Content Validity

Salvador Chacón-Moscoso^*^, Susana Sanduvete-Chaves, M. Teresa Anguera, José L. Losada, Mariona Portell, José A. Lozano-Lozano

*** Correspondence:** Salvador Chacón-Moscoso: schacon@us.es

Appendix I. Content validity questionnaire to delimit the main characteristics in sports-related observational studies (English version)

| Instructions: You are asked to collaborate in a content validity study to delimit the characteristics of sports-related observational studies. Please assess the degree of representativeness, relevance, utility, and feasibility from 1 to 5, with 1 the lowest and 5 being the highest score for each of the items with respect to its dimension: 1) Representativeness is defined as the degree to which each item represents its dimension. 2) Relevance refers to the extent to which the item is important or highlights something of the dimension it represents. 3) Utility refers to the extent to which each specific item is useful for measuring the assigned dimension. 4) Feasibility refers to the extent to which it is possible to record item information. If you consider it appropriate, you may add some additional comments in the final part of the questionnaire in order to improve any item propose a more appropriate item, or remove an existing one. |
| --- |
| Dimension 1. Extrinsic characteristics  **Item 1**. Publication type: source of information from which the work was obtained (Sánchez-Meca and Ato, 1989; Sánchez-Meca, 1997): 1) Journal: periodical scientific publication, in connection with a series of issues or specifications. 2) Book: scientific publication with enough length to cover a volume, in press or in any other format. 3) Thesis: written and presented in a University by those candidates to obtain the degree of doctor. 4) Congress: work presented in a conference, usually periodic, in which members of an association, organization, profession, etc. meet for reasons previously established. |
| Dimension 2. Objectives delimitation |
| **Item 2**. Problem delimitation, sport: 1) Physical activity. 2) Athletics. 3) Basketball. 4) Handball. 5) Boxing. 6) Teaching of physical activity. 7) Climbing. 8) Fencing. 9) Skiing. 10) Soccer. 11) Indoor soccer. 12) Hockey. 13) Judo. 14) Karate. 15) Swimming. 16) Psychomotor skills. 17) Tae kwon do. 18) Tennis. 19) Users of sport services. 20) Volleyball. 21) Others (specify). |
| **Item 3**. Problem delimitation, general objective/s is/are specified: 1) No. 2) Yes. |
| **Item 4**. Problem delimitation, specific objective/s is/are specified: 1) No. 2) Yes. |
| **Item 5**. Reference to theoretical framework: 1) No. 2) Yes, with complete empirical definition of constructs. 3) Yes, with partial empirical definition of constructs. 4) Yes, without empirical definition of constructs. 5) Regulation. |
| **Item 6**. Specification of response level/s considered (Portell et al., 2015): 1) No. 2) Yes. |
| **Item 7**. Specification of the participation degree: 1) No. 2) Not participating. 3) Participating. 4) Participation-observation. 5) Self-observation. 6) Several types (please specify). |
| Dimension 3. Observational design |
| **Item 8**. Specification of the observational design for each specific objective: 1) No. 2) Idiographic/Follow-up/One-dimension. 3) Idiographic/Follow-up/Multidimensional. 4) Idiographic/One-time/one-dimension. 5) Idiographic/One-time/Multidimensional. 6) Nomothetic/Follow-up/One-dimension. 7) Nomothetic/Follow-up/Multidimensional. 8) Nomothetic/One-time/One-dimension. 9) Nomothetic/One-time/Multidimensional. |
| **Item 9**. Justification of the observational design: 1) No.2) Yes. |
| **Item 10**. Are sequential data obtained? (Portell et al., 2015):1) No. 2) Yes. |
| Dimension 4. Participants |
| **Item 11**. Age: 1) Under 6s. 2) Under 8s. 3) Under 10s. 4) Under 12s. 5) Under 14s. 6) Under 16s. 7) Under 18s. 8) Under 19s. 9) 19 or more. |
| **Item 12**. Cultural background specified: 1) No. 2) Yes. |
| **Item 13**. Socio-economic level: 1) Low. 2) Medium. 3) High. |
| **Item 14**. Sport modality: 1) Individual sport. 2) Team sport. |
| **Item 15**. Professionalism: 1) Professionals. 2) Semi-professionals. 3) Sportsmen/women in training stage. |
| **Item 16**. Exclusion/rejection of participants, global (from the beginning to the end across participants): 1) No. 2) Yes. |
| **Item 17**. Exclusion/rejection of participants, differential (from the beginning to the end across groups): 1) No. 2) Yes. |
| **Item 18**. Cancelled participants’ allocation: 1) No. 2) Yes. |
| **Item 19**. Activity type: 1) Training, beginner level. 2) Training, expert or amateur level. 3) Training, elite or professional level. 4) Local competition. 5) Regional competition. 6) National competition. 7) Continental competition. 8) Worldwide competition. |
| Dimension 5. Context (setting) |
| **Item 20**. Place (location): 1) Home game. 2) Away game. 3) Neutral game. |
| **Item 21**. Social/public impact of the activity: 1) No. 2) Yes (competition). |
| **Item 22**. Time frame: indication of the year or season: 1) No. 2) Yes. |
| **Item 23**. Specification of the session’s acceptance criteria: 1) No. 2) Yes, between-session constancy. 3) Yes, within-session constancy. 3) Yes, temporal disruptions. |
| **Item 24**. Number of non-observable periods (in the case they are given): (please insert value). |
| **Item 25**. Average duration of non-observable periods (in the case they are given): (please insert value). |
| **Item 26**. Total results indication: 1) No. 2) Yes. |
| **Item 27**. Partial results indication: 1) No. 2) Yes. |
| **Item 28**. Observational unit’s specificity adjustment: 1) No. 2) According to theoretical framework or regulation. 3) According to activity by play. 4) According to activity by actions (elements of the play). 5) Temporal conventional (parts of the match: halves, quarters, etc.). 6) Temporal established *ad hoc*. |
| **Item 29**. Observational units meet the *3 Ds rule* (delimiting, denominating, and definable): 1) No. 2) Yes. |
| **Item 30**. Global/molecular units’ degree is justified: 1) No. 2) Yes. |
| Dimension 6. Observational instrument |
| **Item 31**. Type of observational instrument: 1) Nominal categories system. 2) Ordinal categories system. 3) Field format. 4) Combination of field format and categories system. 5) Estimation scale. |
| **Item 32**. Appropriateness of the instrument to the observational design: 1) No. 2) Yes. |
| **Item 33**. Justification of instrument type developed according to the observational design presented (one-dimensional vs. multidimensional). 1) No. 2) Yes. |
| **Item 34**. When combining field format and categories systems, specification of the criteria that led to the catalog(s) and the categories system(s):1) No. 2) Yes. |
| **Item 35**. When the criteria that led to the catalog(s) and the categories system(s) are specified, inclusion of the requirements to categorize from a certain criterion: 1) No. 2) Availability of theoretical framework (or official rules). 3) Existence of timelessness. |
| **Item 36**. Availability of full coding manual: 1) No. 2) Yes. |
| **Item37**. Adequacy of the observational instrument to the study context: 1) No. 2) Yes. |
| Dimension 7. Recording instrument |
| **Item 38**. Software utilization as user: 1) No. 2) Yes. |
| **Item 39**. Software type used to record: 1) Free. 2) Paying. 3) Self-developed. |
| **Item 40**. Observational recording: 1) Direct. 2) Indirect. |
| **Item 41**. Software used to record: 1) None (manual recording). 2) SDIS-GSEQ v. 4.2.1./GSEQ 5. 3) MATCH VISION STUDIO/MOTS. 4) LINCE. 5) HOISAN. 6) EYE-SOCCER. 7) LONGOMATCH. 8) Excel. 9) The Observer. 10) Atlas.ti. 11) MaxQDA2. 12) NVivo. 13) Nudist. 14) Transana. 15) Other (specify). |
| **Item 42**. Software used for data quality control: 1) SDIS-GSEQ. 2) LINCE. 3) HOISAN. 4) Other (specify). |
| **Item 43**. Software used for data analysis: 1) SDIS-GSEQ. 2) HOISAN. 3) THEME v.6. 4) Other (specify). |
|  |
| Dimension 8. Data |
| **Item 44**. Type of data according to Bakeman (1978): 1) Type I data. 2) Type II data. 3) Type III data. 4) Type IV data. |
| **Item 45**. Type of data according to Bakeman (1983): 1) Sequential data of event. 2) Sequential data of state. 3) Sequential data of event with time. 4) Sequential data of range. 5) Sequential data of multi-event. |
| **Item 46**. Data management: 1) Differentiation of sessions. 2) Aggregation of sessions. 3) Partition of sessions. |
| Dimension 9. Parameters specification |
| **Item 47**. Parameters type (indicate the most complex):1) Static primary behavioral indicators: frequency. 2) Static primary behavioral indicators: sequence. 3) Static primary behavioral indicators: duration. 4) Static primary behavioral indicators: lapse. 5) Static primary behavioral indicators: latency. 6) Static primary behavioral indicators: intensity. 7) Static secondary or derivative behavioral indicators: rate. 8) Static secondary or derivative behavioral indicators: relative frequency. 9) Static secondary or derivative behavioral indicators: relative duration. 10) Static secondary or derivative behavioral indicators: average duration. 11) Dynamic behavioral indicators: transition frequency. 12) Dynamic behavioral indicators: relative transition frequency. 13) Dynamic behavioral indicators related to sequential structure of the behavior. 14) Dynamic behavioral indicators related to T-patterns detection. |
| **Item 48**. Parameter fitting (indicate the most complex): 1) Modified frequency. 2) Sanson-Fisher modified frequency. 3) Frequency estimation. 4) Duration estimation. 5) Relative duration estimation. |
| Dimension 10. Observational sampling |
| **Item 49**. Observational period *(please specify number of weeks)*. |
| **Item 50**. Session periodicity *(please specify number of days)*. |
| **Item 51**. Number of sessions *(please specify value)*. |
| **Item 52**. Starting session criterion: 1) Behavioral. 2) Chronometric. 3) Mixed. |
| **Item 53**. Ending session criterion specified: 1) No. 2) Yes. |
| **Item 54**. Within-session sampling: 1) Continuous recording. 2) Second level of sampling: *ad lib*. 3) Second level of sampling: of events. 4) Second level of sampling: focal, starting with participant specification. 5) Second level of sampling: focal, rounds duplication. 6) Scan sampling, time points. 7) Scan sampling, distance between points specification. 8) Scan sampling, effectiveness based on distance calculation. 9) Total intervals, length specification. 10) Total intervals, effectiveness based on length calculation. 11) Partial intervals, length specification. 12) Partial intervals, effectiveness based on length calculation. |
| Dimension 11. Data quality control  This dimension in based on two main concepts: reliability and accuracy. |
| **Item 55**. Agreements: 1) Linear correlation coefficient. 2) Ordinal correlation coefficient. 3) Quasi-correlation. |
| **Item 56**. Concordance: 1) Qualitative method: simple concordance with consensus. 2) Qualitative method: mixed concordance with consensus. 3) Quantitative method, frequency: chance without control: F_%A_. 4) Quantitative way, frequency: partially controlled chance: F_A.P_. 5) Quantitative method, frequency: totally controlled chance: F_global_. 6) Quantitative method, order: Feingold coefficient. 7) Quantitative method, duration: Kappa coefficient. 8) Mixed method: sequential concordance. |
| **Item 57**. Within-session reliability: 1) Global, Pearson correlation. 2) Global, Berkintra-class coefficient. 3) Sequential, Pearson correlation. 4) Sequential, Berk intra-class coefficient. 5) Point by point, agreement percentages. 6) Point by point, Kappa coefficient. 7) Point by point, Cohen Kappa. 8) Point by point, Kappa for occurrences. 9) Point by point, Kappa for non-occurrences. 10) Point by point, Pi. 11) Point by point, G. 12) Point by point, Lambda. 13) Point by point, Yule Q. 14) Point by point, Phi. |
| **Item 58**. Between-session reliability: 1) Global, Pearson correlation. 2) Global, Berk intra-class coefficient. 3) Sequential, Pearson correlation. 4) Sequential, Berk intra-class coefficient. 5) Point by point, agreement percentages. 6) Point by point, Kappa coefficient. 7) Point by point, Cohen Kappa. 8) Point by point, Kappa for occurrences. 9) Point by point, Kappa for non-occurrences. 10) Point by point, Pi. 11) Point by point, G. 12) Point by point, Lambda. 13) Point by point, Yule Q. 14) Point by point, Phi. |
| **Item 59**. Generalizability theory application: 1) Scores generalizability: to observe the extent to which data do not depend on the person who gets these data (in psychometrics, it is objectivity or intercoder reliability). 2) Elements generalizability: first, specify the field for which the evaluation is going to be developed. Then, select the test to assess the elements of the set of behaviors to evaluate (in psychometrics, it is content validity or internal consistency). 3) Temporary generalizability: if the data obtained at a particular moment are repeated in other moments, you can say there is test stability and/or you can ensure the possibility of generalizing the data to a temporary universe (in psychometrics, test stability). 4) Situation generalizability: data obtained in artificial contexts can be generalizable to the participant’s real life. This implies checking whether a subject acts in an equal or similar way in different situations (in psychometrics, ecological validity). 5) Methods generalizability: check to what extent the data obtained through different procedures are convergent in a participant (in psychometrics, convergent validity). 6) Dimension generalizability: implies knowing to what extent a series of behavior is associated with a specific case or whether different response modalities are related. 7) Others (specify). |
| Dimension 12. Data analysis |
| **Item 60**. Data analysis developed: 1) Descriptive analysis. 2) Relationship between categorical data. 3) Odd ratios. 4) Search for regularities, sequential analysis of delays. 5) Search for regularities, Markov chains. 6) Search for regularities, T-patterns detection. 7) Search for regularities, analysis of polar coordinates. 8) Behavior vectorization. 9) Multivariate analysis, logistic regression. 10) Multivariate analysis, log-linear. 11) Multivariate analysis, logit-probit. 12) Multivariate analysis, correspondence analysis. 13) Temporary dimension, trends analysis. 14) Temporary dimension, time series. 15) Temporary dimension, panel studies. 16) Non-parametric tests. 17) Relationship test, ordinal correlation. 18) Relationship test, linear correlation. 19) Relationship test, multiple correlation. 20) Multidimensional scaling. |
| COMMENTS: |

*Note*: Encode with a 99 when data are not provided or the item is not applicable.

Appendix II. Final version of the checklist for reporting sports-related observational studies after the content validity study

Please mark the corresponding option.

| Dimension 1. Extrinsic characteristic  **Item 1**. Publication type: source of information from which the work was obtained (Sánchez-Meca and Ato, 1989; Sánchez-Meca, 1997): 1) Journal: periodical scientific publication, in connection with a series of issues or specifications. 2) Book: scientific publication, with enough length to cover a volume, in press or in any other format. 3) Thesis: written and presented in a University by those candidates to obtain the degree of doctor. 4) Congress: work presented in a conference, usually periodic, in which members of an association, organization, profession, etc. meet for reasons previously established. |
| --- |
| Dimension 2. Objectives delimitation |
| **Item 2**. Problem delimitation, sport: 1) Physical activity. 2) Athletics. 3) Basketball. 4) Handball. 5) Boxing. 6) Teaching of physical activity. 7) Climbing. 8) Fencing. 9) Skiing. 10) Soccer. 11) Indoor soccer (12) Hockey. 13) Judo. 14) Karate. 15) Swimming. 16) Modified/reduced sport games. 17) Taekwondo. 18) Tennis. 19) Users of sport services. 20) Volleyball. 21) Others (specify). |
| **Item 3**. Problem delimitation, general objective/s is/are specified: 1) No. 2) Yes, but unclearly. 3) Yes. |
| **Item 4**. Problem delimitation, specific objective/s is/are specified: 1) No. 2) Yes, but unclearly. 3) Yes. |
| **Item 5**. Reference to theoretical framework: 1) No. 2) Yes, with complete empirical definition of constructs. 3) Yes, with partial empirical definition of constructs. 4) Yes, without empirical definition of constructs. |
| **Item 6**. Specification of response level/s considered (Portell et al., 2015): 1) No. 2) Yes, but unclearly. 3) Yes. |
| **Item 7**. Specification of the participation degree: 1) No. 2) Not participating. 3) Participating. 4) Participation-observation. 5) Self-observation. 6) Several types (please, specify). |
| Dimension 3. Observational design |
| **Item 8**. Specification of the observational design for each specific objective: 1) No. 2) Idiographic/Follow-up/One-dimension. 3) Idiographic/Follow-up/Multidimensional. 4) Idiographic/One-time/one-dimensional. 5) Idiographic/One-time/Multidimensional. 6) Nomothetic/Follow-up/One-dimension. 7) Nomothetic/Follow-up/Multidimensional. 8) Nomothetic/One-time/One-dimension. 9) Nomothetic/One-time/Multidimensional. |
| **Item 9**. Justification of the observational design: 1) No. 2) Partial/incomplete; 3) Yes. |
| **Item 10**. Are sequential data obtained? (Portell et al. 2015): 1) No. 2) Yes. |
| Dimension 4. Participants |
| **Item 11**. Age: 1) Under 6s. 2) Under 8s. 3) Under 10s. 4) Under 12s. 5) Under 14s. 6) Under 16s. 7) Under 18s. 8) Under 19s. 9) Under 20s; 20 or more. |
| **Item 12**. Sport modality: 1) Individual sport. 2) Team sport.  **Item 13**. Sport with opposition: 1) No. 2) Yes. |
| **Item 14**. Professionalism: 1) Amateur. 2) Sportsmen/women in training stage. 3) Semi-professionals. 4) Professionals. 5) Retired from professional sport. |
| **Item 15**. Activity type: 1) Training, beginner level. 2) Training, expert or amateur level. 3) Training, elite or professional level. 4) Local competition. 5) Regional competition. 6) National competition. 7) Continental competition. 8) Worldwide competition. 9) Physical exercise. |
| Dimension 5. Context (setting) |
| **Item 16**. Place (location): 1) Home game. 2) Away game. 3) Neutral game. |
| **Item 17**. Social/public impact of the activity: 1) Low. 2) Medium. 3) High (competition). |
| **Item 18**. Time frame: indication of the year or season: 1) No. 2) Yes. |
| **Item 19**. Specification of the session’s acceptance criteria: 1) No. 2) Yes, between-session constancy. 3) Yes, within-session constancy. 3) Yes, temporal disruptions. |
| **Item 20**. Average duration of non-observable periods (in the case they are given): (please insert value). |
| **Item 21**. Total results indication: 1) No. 2) Yes. |
| **Item 22**. Partial results indication: 1) No. 2) Yes. |
| **Item 23**. Observational unit’s specificity adjustment: 1) No. 2) According to theoretical framework or regulation. 3) According to activity by play. 4) According to activity by actions (elements of the play). 5) Temporal conventional (parts of the match: halves, quarters, etc.). 6) Temporal established *ad hoc*. 7) According to the player. |
| **Item 24**. Observational units meet the *3 Ds rule* (delimiting, denominating, and definable): 1) No. 2) Yes. |
| **Item 25**. Global/molecular units’ degree is justified: 0) No. 1) Yes. |
| Dimension 6. Observational instrument |
| **Item 26**. Type of observational instrument: 1) Nominal categories system. 2) Ordinal categories system. 3) Field format. 4) Combination of field format and categories system. 5) Estimation scale. |
| **Item 27**. Appropriateness of the instrument to the observational design: 1) No. 2) Yes. |
| **Item 28**. Justification of instrument type developed according to the observational design presented (one-dimensional vs. multidimensional). 1) No. 2) Yes. |
| **Item 29**. When the criteria that led to the catalog(s) and the categories system(s) are specified, inclusion of the requirements to categorize from a certain criterion: 1) No. 2) Availability of theoretical framework (or official rules). 3) Existence of timelessness. |
| **Item 30**. Availability of full coding manual: 1) No. 2) Yes. |
| **Item 31**. Adequacy of the observational instrument to the context of study: 1) No. 2) Partial. 3) Yes. |
| Dimension 7. Recording instrument |
| **Item 32**. Software utilization as user: 1) No. 2) Yes. |
| **Item 33**. Software type used to record: 1) Free. 2) Paying. 3) Self-developed. |
| **Item 34**. Observational recording: 1) Direct. 2) Indirect. |
| **Item 35**. Software used to record: 1) None (manual recording). 2) SDIS-GSEQ v. 4.2.1./GSEQ 5. 3) MATCH VISION STUDIO/MOTS. 4) LINCE. 5) HOISAN. 6) EYE-SOCCER. 7) LONGOMATCH. 8) Excel. 9) The Observer. 10) Atlas.ti. 11) MaxQDA2. 12) NVivo. 13) Nudist. 14) Transana. 15) SportsCode. 16) Other (specify). |
|  |
|  |
| Dimension 8. Data |
| **Item 36**. Type of data according to Bakeman (1978): 1) Type I data. 2) Type II data. 3) Type III data. 4) Type IV data. |
| **Item 37**. Type of data according to Bakeman (1983): 1) Sequential data of event. 2) Sequential data of state. 3) Sequential data of event with time. 4) Sequential data of range. 5) Sequential data of multi-event. |
| **Item 38**. Data management: 1) Differentiation of sessions. 2) Aggregation of sessions. 3) Partition of sessions. |
| Dimension 9. Parameter specification |
| **Item 39**. Parameter type (indicate the most complex): 1) Static primary behavioral indicators: frequency. 2) Static primary behavioral indicators: sequence. 3) Static primary behavioral indicators: duration. 4) Static primary behavioral indicators: lapse. 5) Static primary behavioral indicators: latency. 6) Static primary behavioral indicators: intensity. 7) Static secondary or derivative behavioral indicators: rate. 8) Static secondary or derivative behavioral indicators: relative frequency. 9) Static secondary or derivative behavioral indicators: relative duration. 10) Static secondary or derivative behavioral indicators: average duration. 11) Dynamic behavioral indicators: transition frequency. 12) Dynamic behavioral indicators: relative transition frequency. 13) Dynamic behavioral indicators related to sequential structure of the behavior. 14) Dynamic behavioral indicators related to T-pattern detection. |
|  |
| Dimension 10. Observational sampling |
| **Item 40**. Observational period *(please specify number of weeks)*. |
| **Item 41**. Sessions periodicity *(please specify number of days)*. |
| **Item 42**. Number of sessions *(please specify value)*. |
| **Item 43**. Starting session criterion: 1) Behavioral. 2) Chronometric. 3) Mixed. |
| **Item 44**. Ending session criterion specified: 1) No. 2) Yes. |
| **Item 45**. Within-session sampling: 1) Continuous recording. 2) Second level of sampling: *ad lib*. 3) Second level of sampling: of events. 4) Second level of sampling: focal, starting participant specification. 5) Second level of sampling: focal, rounds duplication. 6) Scan sampling, time points. 7) Scan sampling, distance between points specification. 8) Scan sampling, effectiveness based on distance calculation. 9) Total intervals, length specification. 10) Total intervals, effectiveness based on length calculation. 11) Partial intervals, length specification. 12) Partial intervals, effectiveness based on length calculation. |
| Dimension 11. Data quality control |
| **Item 46**. Software used for data quality control: 1) SDIS-GSEQ. 2) LINCE. 3) HOISAN. 4) Other (specify). |
| **Item 47**. Exclusion/rejection of participants, global (from the beginning to the end across participants): 1) No. 2) Yes. |
| **Item 48**. Agreements: 1) Linear correlation coefficient. 2) Ordinal correlation coefficient. 3) Quasi-correlation. |
| **Item 49**. Concordance: 1) Qualitative method: simple concordance with consensus. 2) Qualitative method: mixed concordance with consensus. 3) Quantitative method, frequency: chance without control: F_%A_. 4) Quantitative method, frequency: partially controlled chance: F_A.P_. 5) Quantitative method, frequency: totally controlled chance: F_global_. 6) Quantitative method, order: Feingold coefficient. 7) Quantitative method, duration: Kappa coefficient. 8) Mixed method: sequential concordance. |
| **Item 50**. Within-session reliability: 1) Global, Pearson correlation. 2) Global, Berk intra-class coefficient. 3) Sequential, Pearson correlation. 4) Sequential, Berk intra-class coefficient. 5) Point by point, agreement percentages. 6) Point by point, Kappa coefficient. 7) Point by point, Cohen Kappa. 8) Point by point, Kappa for occurrences. 9) Point by point, Kappa for non-occurrences. 10) Point by point, Pi. 11) Point by point, G. 12) Point by point, Lambda. 13) Point by point, Yule Q. 14) Point by point, Phi. |
| **Item 51**. Between-session reliability: 1) Global, Pearson correlation. 2) Global, Berk intra-class coefficient. 3) Sequential, Pearson correlation. 4) Sequential, Berk intra-class coefficient. 5) Point by point, agreement percentages. 6) Point by point, Kappa coefficient. 7) Point by point, Cohen Kappa. 8) Point by point, Kappa for occurrences. 9) Point by point, Kappa for non-occurrences. 10) Point by point, Pi. 11) Point by point, G. 12) Point by point, Lambda. 13) Point by point, Yule Q. 14) Point by point, Phi. |
| **Item 52**. Generalizability theory application: 1) Scores generalizability: to observe the extent to which data do not depend on the person who gets these data (in psychometrics, it is objectivity or intercoder reliability). 2) Element generalizability: first, specify the field for which the evaluation is going to be developed. Then, you select the test to assess the elements of the set of behaviors that we want to evaluate (in psychometrics, it is content validity or internal consistency). 3) Temporary generalizability: if the data obtained at a particular moment are repeated in other moments, you can say there is test stability and/or you can ensure the possibility of generalizing the data to a temporary universe (in psychometrics, test stability). 4) Situation generalizability: data obtained in artificial contexts can be generalizable to the participant’s real life. This implies checking whether a subject acts in an equal or similar way in different situations (in psychometrics, ecological validity). 5) Method generalizability: check to what extent the data obtained through different procedures are convergent in a participant (in psychometrics, convergent validity). 6) Dimension generalizability: this implies knowing to what extent a series of behavior is associated with a specific case or whether different response modalities are related. 7) Others (specify). |
| Dimension 12. Data analysis |
| **Item 53**. Software used for data analysis: 1) SDIS-GSEQ. 2) HOISAN. 3) THEME v.6. 4) Other (specify). |
| **Item 54**. Data analysis developed: 1) Descriptive analysis. 2) Relationship between categorical data. 3) Odd ratios. 4) Search for regularities, sequential analysis of delays. 5) Search for regularities, Markov chains. 6) Search for regularities, T-Patterns detection. 7) Search for regularities, analysis of polar coordinates. 8) Behavior vectorization. 9) Multivariate analysis, logistic regression. 10) Multivariate analysis, log-linear. 11) Multivariate analysis, logit-probit. 12) Multivariate analysis, correspondence analysis. 13) Temporary dimension, trends analysis. 14) Temporary dimension, time series. 15) Temporary dimension, studies of panel. 16) Non-parametric tests. 17) Relationship test, ordinal correlation. 18) Relationship test, linear correlation. 19) Relationship test, multiple correlation. 20) Multidimensional scaling. |

*Note*: Encode with a 99 when data are not provided or the item is not applicable.

**References**

Bakeman, R. (1978). Untangling streams of behavior: Sequential analysis of observation data. In G. P. Sackett (Ed.), *Observing behavior, vol. 2: Data collection and analysis methods* (pp. 63–78). Baltimore, MD: University of Park Press.

Bakeman, R. (1983). Computing lag sequential analysis statistics: The ELAG program. *Behavior Research, Methods* & *Instruments, 15*, 530–535. doi:10.3758/BF03203700

Portell, M., Anguera, M. T., Chacón, S., & Sanduvete, S. (2015). Guidelines for reporting evaluations based on observational methodology. *Psicothema, 27*(3), 283–289. doi:10.7334/psicothema2014.276

Sánchez-Meca, J. (1997). Methodological issues in the meta-evaluation of correctional treatment. In S. Redondo, V. Garrido, J. Pérez, & R. Barberet (Ed.), *Advances in psychology and law. International contributions* (pp. 486–498). New York, NY: Walter de Gruyter.

Sánchez-Meca, J., & Ato, M. (1989). Meta-análisis: una alternativa metodológica a las revisiones tradicionales de la investigación [Meta-analysis: A methodological alternative to traditional research reviews]. In J. Arnau, & H. Carpintero (Eds.), *Tratado de psicología general* (pp. 617–669). Madrid, Spain: Alhambra.
