# Supplementary material for: Preliminary Checklist for Reporting Observational Studies in Sports Areas: Content Validity
Source: Front Psychol. 2018 Mar 8;9:291. doi: 10.3389/fpsyg.2018.00291 (PMC5853306; doi:10.3389/fpsyg.2018.00291)
Supplement: Supplementary file 2 [file Table2.docx]

Supplementary Material

Preliminary Checklist for Reporting Observational Studies in Sports Areas: Content Validity

Salvador Chacón-Moscoso^*^, Susana Sanduvete-Chaves, M. Teresa Anguera, José L. Losada, Mariona Portell, José A. Lozano-Lozano

*** Correspondence:** Salvador Chacón-Moscoso: schacon@us.es

# Supplementary Table. Content validity questionnaire to delimit the main characteristics in sports-related observational studies (Spanish version)

| Instrucciones. Se le solicita su colaboración para participar en un estudio de validez de contenido, para acotar las características metodológicas en estudios observacionales sobre deporte. Por favor, valore de 1 a 5 el grado de representatividad, relevancia, utilidad y viabilidad, siendo 1 el grado más bajo y 5 el más alto, de cada uno de los ítems respecto a su dimensión: 1) Representatividad: grado en que cada ítem representa a su dimensión. 2) Relevancia: en qué medida el ítem es importante o destaca algo de la dimensión que representa. 3) Utilidad: en qué medida cada ítem específico es útil para medir la dimensión asignada. 4) Viabilidad: en qué medida es posible registrar la información del ítem. Si lo considera oportuno, en la parte final del cuestionario puede realizar algún comentario adicional para mejorar cualquier ítem e incluso proponer un ítem más adecuado o eliminar alguno de los existentes. |
| --- |
| Dimensión 1. Característica extrínseca  **Ítem 1**. Tipo de publicación: fuente de información de la que proviene el trabajo que se evalúa (Sánchez-Meca & Ato, 1989; Sánchez-Meca, 1997): 1) Revista: publicación científica periódica, en relación con una serie de cuestiones o especificaciones. 2) Libro: publicación científica, con suficiente longitud para cubrir un volumen, en prensa o en cualquier otro formato. 3) Tesis: escrita y presentada en una universidad por los candidatos al grado de doctor de una facultad. 4) Congreso: trabajo presentado en una conferencia generalmente periódica, en el que los miembros de una asociación, organización, profesión, etc. se reúnen entre sí para asuntos previamente establecidos. |
| Dimensión 2. Delimitación de objetivos |
| **Ítem 2**. Acotación del problema, deporte: 1) Actividad física. 2) Atletismo. 3) Baloncesto. 4) Balonmano. 5) Boxeo. 6) Enseñanza de actividad física. 7) Escalada. 8) Esgrima. 9) Esquí. 10) Fútbol. 11) Fútbol-sala. 12) Hockey. 13) Judo. 14) Karate. 15) Natación. 16) Psicomotricidad. 17) Taekwondo. 18) Tenis. 19) Usuarios de servicios deportivos. 20) Voleibol. 21) Otros (especificar). |
| **Ítem 3**. Acotación del problema, objetivo/s general/es especificado/s: 1) No. 2) Sí. |
| **Ítem 4**. Acotación del problema, objetivo/s específico/s especificado/s 1) No. 2) Sí. |
| **Ítem 5**. Referencia a marco teórico: 1) No. 2) Sí, con constructos completamente definidos de manera operativa. 3) Sí, con constructos parcialmente definidos de manera operativa. 4) Sí, sin constructos definidos operativamente. 5) Reglamento. |
| **Ítem 6**. Especificación del nivel o niveles de respuesta considerados (Portell et al., 2015): 1) No. 2) Sí. |
| **Ítem 7**. Especificación del grado de participación: 1) No. 2) No participante. 3) Participante. 4) Participación-observación. 5) Auto-observación. 6) Varios Tipos (especificar). |
| Dimensión 3. Diseño observacional |
| **Ítem 8**. Especificación del diseño observacional para cada objetivo específico: 1) No. 2) Ideográfico / Seguimiento / Unidimensional. 3) Ideográfico / Seguimiento / Multidimensional. 4) Ideográfico / Puntual/ Unidimensional. 5) Ideográfico/ Puntual/ Multidimensional. 6) Nomotético/ Seguimiento/ Unidimensional. 7) Nomotético / Seguimiento / Multidimensional. 8) Nomotético / Puntual / Unidimensional. 9) Nomotético / Puntual / Multidimensional. |
| **Ítem 9**. Justificación del diseño observacional: 1) No. 2) Sí. |
| **Ítem 10**. ¿Se obtienen datos secuenciales? (Portell et al., 2015): 1) No. 2) Sí. |
| Dimensión 4. Participantes |
| **Ítem 11**. Edad: 1) Menores de pre-benjamín: sub-6. 2) Pre-benjamín: sub-7 y sub-8. 3) Benjamín: sub-9 y sub-10. 4) Alevín: sub-11 y sub-12. 5) Infantil: sub-13 y sub-14. 6) Cadete: sub-15 y sub-16. 7) Juvenil: sub-17 y sub-18. 8) Juvenil de tercer año: sub-19. 9) Absoluto: sénior. |
| **Ítem 12**. Antecedentes culturales especificados: 1) No. 2) Sí. |
| **Ítem 13**. Nivel socioeconómico: 1) Bajo. 2) Medio. 3) Alto. |
| **Ítem 14**. Modalidad deportiva: 1) Deporte individual. 2) Deporte de equipo. |
| **Ítem 15**. Profesionalidad: 1) Deportistas profesionales. 2) Semi-profesional. 3) Deportistas en fase de formación. |
| **Ítem 16**. Exclusión / rechazo de participantes global (de inicio a fin entre participantes): 1) No. 2) Sí. |
| **Ítem 17**. Exclusión / rechazo de participantes diferencial (de inicio a fin entre grupos): 1) No. 2) Sí. |
| **Ítem 18**. Des-asignación de participantes: 1) No. 2) Sí. |
| **Ítem 19**. Tipo de actividad: 1) Entrenamiento, nivel principiante. 2) Entrenamiento, nivel experto o amateur. 3) Entrenamiento, nivel élite o profesional. 4) Competición local. 5) Competición autonómica. 6) Competición nacional. 7) Competición continental. 8) Competición mundial. |
| Dimensión 5. Contexto |
| **Ítem 20**. Lugar (emplazamiento): 1) Jugar *en casa*. 2) Jugar *en casa del adversario*. 3) Campo neutral. |
| **Ítem 21**. Incidencia pública/social de la actividad: 1) No. 2) Sí (competición). |
| **Ítem 22**. Marco temporal: indicación del año o temporada: 1) No. 2) Sí. |
| **Ítem 23**. Especificación de los criterios de aceptación de sesiones: 1) No. 2) Sí, constancia inter-sesional. 3) Sí, constancia intra-sesional. 4) Sí, disrupciones temporales. |
| **Ítem 24**. Número de períodos de inobservabilidad (en su caso): (introducir valor)*.* |
| **Ítem 25**. Duración media de períodos de inobservabilidad (en su caso): (introducir valor)*.* |
| **Ítem 26**. Indicación de resultados totales: 1) No. 2) Sí. |
| **Ítem 27**. Indicación de resultados parciales: 1) No. 2) Sí. |
| **Ítem 28**. Ajuste de la especificación de unidades de observación: 1) No. 2) Según marco teórico o reglamento. 3) Según actividad por jugadas. 4) Según actividad por acciones (elementos de la jugada). 5) Temporales convencionales (partes del partido: mitades, cuartos, etc.). 6) Temporales establecidas *ad hoc*. |
| **Ítem 29**. Las unidades de observación cumplen la “regla de las 3 D” (delimitable, denominable y definible): 1) No. 2) Sí. |
| **Ítem 30**. El grado de molaridad / molecularidad de las unidades está justificado: 1) No. 2) Sí. |
| Dimensión 6. Instrumento de observación |
| **Ítem 31**. Tipo de instrumento de observación: 1) Sistema de categorías nominal. 2) Sistema de categorías ordinal. 3) Formato de campo. 4) Combinación entre formato de campo y sistema de categorías. 5) Escala de estimación. |
| **Ítem 32**. Adecuación del instrumento al diseño observacional planteado: 1) No. 2) Sí. |
| **Ítem 33**. Justificación del tipo de instrumento construido según el diseño observacional planteado (unidimensional vs. multidimensional): 1) No. 2) Sí. |
| **Ítem 34**. En los casos de combinación entre formato de campo y sistemas de categorías, especificación de los criterios que dan lugar al (a los) catálogo(s) y al (a los) sistema(s) de categorías: 1) No. 2) Sí. |
| **Ítem 35**. En caso de especificación de los criterios que dan lugar al (a los) catálogos y al (a los) sistema(s) de categorías, inclusión de los requisitos para categorizar a partir de un determinado criterio: 1) No. 2) Disponibilidad de marco teórico (o reglamento oficial). 3) Existencia de atemporalidad. |
| **Ítem 36**. Disponibilidad del manual de codificación completo: 1) No. 2) Sí. |
| **Ítem 37**. Adecuación del instrumento de observación al contexto de estudio: 1) No. 2) Sí. |
| Dimensión 7. Instrumento de registro |
| **Ítem 38**. Utilización de software como usuario: 1) No. 2) Sí. |
| **Ítem 39**. Tipo de software utilizado para registrar: 1) Abierto. 2) Comercial. 3) Autoelaborado. |
| **Ítem 40**. Registro de observación: 1) Directa. 2) Indirecta. |
| **Ítem 41**. Software utilizado para registrar: 1) Ninguno (registro manual). 2) SDIS-GSEQ v. 4.2.1. / GSEQ 5. 3) MATCH VISION STUDIO / MOTS. 4) LINCE. 5) HOISAN. 6) EYE-SOCCER. 7) LONGOMATCH. 8) Excel. 9) The Observer. 10) Atlas.ti. 11) MaxQDA2. 12) NVivo. 13) Nudist. 14) Transana. 15) Otro (indicar). |
| **Ítem 42**. Software utilizado para control de calidad del dato: 1) SDIS-GSEQ. 2) LINCE. 3) HOISAN. 4) Otro (especificar). |
| **Ítem 43**. Software utilizado para el análisis de datos: 1) SDIS-GSEQ. 2) HOISAN. 3) THEME v.6. 4) Otro (especificar). |
| Dimensión 8. Datos |
| **Ítem 44**. Tipo de datos según Bakeman (1978): 1) Datos tipo I. 2) Datos tipo II. 3) Datos tipo III. 4) Datos tipo IV. |
| **Ítem 45**. Tipo de datos según Bakeman (1983): 1) Datos secuenciales de evento. 2) Datos secuenciales de estado. 3) Datos secuenciales de evento con tiempo. 4) Datos secuenciales de intervalo. 5) Datos secuenciales de multievento. |
| **Ítem 46**. Gestión de datos: 1) Diferenciación de sesiones. 2) Agregación de sesiones. 3) Partición de sesiones.  Dimensión 9. Especificación de parámetros |
| **Ítem 47**. Tipo de parámetros (indicar el más complejo): 1) Indicadores conductuales estáticos primarios: frecuencia. 2) Indicadores conductuales estáticos primarios: secuencia. 3) Indicadores conductuales estáticos primarios: duración. 4) Indicadores conductuales estáticos primarios: lapso. 5) Indicadores conductuales estáticos primarios: latencia. 6) Indicadores conductuales estáticos primarios: intensidad. 7) Indicadores conductuales estáticos secundarios o derivados: tasa. 8) Indicadores conductuales estáticos secundarios o derivados: frecuencia relativa. 9) Indicadores conductuales estáticos secundarios o derivados: duración relativa. 10) Indicadores conductuales estáticos secundarios o derivados: duración media. 11) Indicadores conductuales dinámicos: frecuencia de transición. 12) Indicadores conductuales dinámicos: frecuencia de transición relativa. 13) Indicadores conductuales dinámicos relacionados con la estructura secuencial de la conducta. 14) Indicadores conductuales dinámicos relacionados con la detección de *T-patterns*. |
| **Ítem 48**. Ajuste de parámetros (indicar el más complejo): 1) Frecuencia modificada. 2) Frecuencia modificada de Sanson-Fisher. 3) Estimación de la frecuencia. 4) Estimación de la duración. 5) Estimación de la duración relativa.  Dimensión 10. Muestreo observacional |
| **Ítem 49**. Período de observación *(especificar número de semanas).* |
| **Ítem 50**. Periodicidad de las sesiones *(introducir número en días).* |
| **Ítem 51**. Número de sesiones *(introducir valor).* |
| **Ítem 52**. Criterio de inicio de sesión: 1) Comportamental. 2) Cronométrico. 3) Mixto. |
| **Ítem 53**. Criterio de fin de sesión especificado: 1) No. 2) Sí. |
| **Ítem 54**. Muestreo intrasesional: 1) Registro continuo. 2) Segundo nivel de muestreo: *ad libitum*. 3) Segundo nivel de muestreo: de eventos. 4) Segundo nivel de muestreo: focal, especificación del participante inicial. 5) Segundo nivel de muestreo: focal, duplicación de rondas. 6) Muestreo *scan,* puntos de tiempo. 7) Muestreo *scan,* especificación distancia entre puntos. 8) Muestreo *scan,* cálculo de la eficacia según distancia. 9) Intervalos totales, especificación de la longitud. 10) Intervalos totales, cálculo de la eficacia según longitud. 11) Intervalos parciales, especificación de la longitud. 12) Intervalos parciales, cálculo de la eficacia según longitud. |
| Dimensión 11. Control de calidad del dato  Se centra en dos conceptos fundamentalmente: la fiabilidad y la precisión. |
| **Ítem 55**. Acuerdos: 1) Coeficiente de correlación lineal. 2) Coeficiente de correlación ordinal. 3) Cuasi-correlación. |
| **Ítem 56**. Concordancia: 1) Forma cualitativa: concordancia consensuada simple. 2) Forma cualitativa: concordancia consensuada mixta. 3) Forma cuantitativa, frecuencia: no control del azar: F_%A_. 4) Forma cuantitativa, frecuencia: control parcial del azar: F_A.P_. 5) Forma cuantitativa, frecuencia: control total del azar: F_global_. 6) Forma cuantitativa, orden: coeficiente de Feingold. 7) Forma cuantitativa, duración: coeficiente Kappa. 8) Forma mixta: concordancia secuencial. |
| **Ítem 57**. Fiabilidad intra-sesional: 1) Global, correlación de Pearson. 2) Global, coeficiente intra-clase de Berk. 3) Secuencial, correlación de Pearson. 4) Secuencial, coeficiente intra-clase de Berk. 5) Punto por punto, porcentajes de acuerdo. 6) Punto por punto, coeficiente Kappa. 7) Punto por punto, Kappa de Cohen. 8) Punto por punto, Kappa para ocurrencias. 9) Punto por punto, Kappa para no ocurrencias. 10) Punto por punto, Pi. 11) Punto por punto, G. 12) Punto por punto, Lambda. 13) Punto por punto, Q de Yule. 14) Punto por punto, Phi. |
| **Ítem 58**. Fiabilidad inter-sesional: 1) Global, correlación de Pearson. 2) Global, coeficiente intra-clase de Berk. 3) Secuencial, correlación de Pearson. 4) Secuencial, coeficiente intra-clase de Berk. 5) Punto por punto, porcentajes de acuerdo. 6) Punto por punto, coeficiente Kappa. 7) Punto por punto, Kappa de Cohen. 8) Punto por punto, Kappa para ocurrencias. 9) Punto por punto, Kappa para no ocurrencias. 10) Punto por punto, Pi. 11) Punto por punto, G. 12) Punto por punto, Lambda. 13) Punto por punto, Q de Yule. 14) Punto por punto, Phi. |
| **Ítem 59**. Aplicación de la teoría de la generalizabilidad: 1) Generalizabilidad de las puntuaciones: se pretende constatar en qué medida los datos no dependen de la persona que los obtiene (en psicometría, objetividad o fiabilidad interjueces). 2) Generalizabilidad de los elementos: primero se debe especificar el ámbito al cual se va a dirigir la evaluación. Después, se selecciona la prueba que evaluará los elementos del conjunto de conductas que queremos evaluar (en psicometría, validez de contenido o consistencia interna). 3) Generalizabilidad temporal: si los datos obtenidos en un momento concreto se repiten en otros momentos, se puede hablar de estabilidad del test y/o se puede asegurar la posibilidad de generalizar los datos a un universo temporal (en psicometría, estabilidad del test). 4) Generalizabilidad de las situaciones: los datos obtenidos en ambientes artificiales pueden ser generalizables en la vida real del sujeto. Se trata de comprobar o verificar si un sujeto actúa de manera igual o similar en diferentes situaciones (en psicometría, validez ecológica). 5) Generalizabilidad de los métodos: se quiere comprobar hasta qué punto los datos obtenidos a través de distintos procedimientos son convergentes entre sí en el sujeto explorado (en psicometría, validez convergente). 6) Generalizabilidad de las dimensiones: se trata de conocer en qué medida una serie de conductas está asociada a un caso concreto o si distintas modalidades de respuesta se relacionan entre sí. 7) Otros (especificar). |
| Dimensión 12. Análisis de datos |
| **Ítem 60**. Análisis realizado: 1) Análisis descriptivo. 2) Relación entre datos categóricos. 3) Comparación de proporciones. 4) Búsqueda de regularidades, análisis secuencial de retardos. 5) Búsqueda de regularidades, cadenas de Markov. 6) Búsqueda de regularidades, detección de T-Patterns. 7) Búsqueda de regularidades, análisis de coordenadas polares. 8) Vectorización del comportamiento. 9) Análisis multivariante, regresión logística. 10) Análisis multivariante, Log-lineal. 11) Análisis multivariante, logit-probit. 12) Análisis multivariante, análisis de correspondencias. 13) Dimensión temporal, análisis de tendencias. 14) Dimensión temporal, series temporales. 15) Dimensión temporal, estudios de panel.16) Pruebas no paramétricas. 17) Pruebas de relación, correlación ordinal. 18) Pruebas de relación, correlación lineal. 19) Pruebas de relación, correlación múltiple. 20) Escalamiento multidimensional. |
| COMENTARIOS: |

*Nota*: codificar con un 99 cuando no se aporte el dato o no proceda.

**References**

Bakeman, R. (1978). Untangling streams of behavior: Sequential analysis of observation data. In G. P. Sackett (Ed.), *Observing behavior, vol. 2: Data collection and analysis methods* (pp. 63–78). Baltimore, MD: University of Park Press.

Bakeman, R. (1983). Computing lag sequential analysis statistics: The ELAG program. *Behavior Research, Methods* & *Instruments, 15*, 530–535. doi:10.3758/BF03203700

Portell, M., Anguera, M. T., Chacón, S., & Sanduvete, S. (2015). Guidelines for reporting evaluations based on observational methodology. *Psicothema, 27*(3), 283–289. doi:10.7334/psicothema2014.276

Sánchez-Meca, J. (1997). Methodological issues in the meta-evaluation of correctional treatment. In S. Redondo, V. Garrido, J. Pérez, & R. Barberet (Ed.), *Advances in psychology and law. International contributions* (pp. 486–498). New York, NY: Walter de Gruyter.

Sánchez-Meca, J., & Ato, M. (1989). [Meta-análisis: una alternativa metodológica a las revisiones tradicionales de la investigación](http://www.um.es/metaanalysis/pdf/6201.pdf) [Meta-analysis: A methodological alternative to traditional research reviews]. In J. Arnau, & H. Carpintero (Eds.), *Tratado de psicología general* (pp. 617–669). Madrid, Spain: Alhambra.
